# Surgical Treatment of Sacroiliac Pigment Villous Nodular Synovitis: A Case Report and Literature Review

**DOI:** 10.3389/fsurg.2022.760704

**Published:** 2022-04-28

**Authors:** Jiashi Song, Kunpeng Jiang, Zhanhu Lv, Bing Liu

**Affiliations:** ^1^Department of Orthopedics, Second Affiliated Hospital, School of Medicine, Zhejiang University, Hangzhou, China; ^2^Department of Orthopedics, Rongjun Hospital, Jiaxing, China; ^3^Department of Orthopedics, Fourth Affiliated Hospital, School of Medicine, Shihezi University, Aksu, China

**Keywords:** sacroiliac joint, clinical manifestations, differential diagnosis, surgical treatment, invasive anal cancer, invasive analysis, the prognosis, pigmented villonodular synovitis

## Abstract

Pigmented villonodular synovitis (PNVS) is a rare and disabling disease that is thought to occur mostly in the knee joint. Here, the authors first present a unique case of PNVS occurring at the sacroiliac joint. The patient complained of sacroiliac joint pain with mild swelling. CT and MRI showed that the tumor was ~63 by 91 by 107 mm in size, and was considered to be a fibrous borderline or low-grade malignancy. Intraoperative macroscopic features of the synovitis during operation suggested PNVS, which was confirmed by histopathological examination. The clinical symptoms and signs of the disease, in this case, are not obvious, and radiological investigations, including MRI, suggest high aggressiveness. The author believes that it may be more likely to relapse and metastasis and recommends complete removal of the synovial membrane and regular follow-up, while preoperative or postoperative radiotherapy and molecular targeted therapy are not recommended for the time being.

## Introduction

Pigmented villonodular synovitis (PVNS) is also known as diffuse giant cell tumor of the tendon sheath, and the main difference from local giant cell tumor of tendon sheath is: the localized type is a benign tumor with a low recurrence rate (10–20%) (1), most commonly occurring in the small joints of fingers or wrists; the diffuse type is most common in knee joint, followed by hip joint, and related cases have also been reported by scholars in the ankle, elbow, and shoulder joint, which is invasive and has a high recurrence rate (20–50%) (2). As a kind of uncommon tumor, the incidence of pigmentation villi nodular synovitis is 1.8/106 (3) according to foreign reports, and the onset age is between 20 to 40 years old, among which female incidence is relatively higher (about 3:1). Also, there have been no relevant reports on the related cases of pigmentation villi nodular sacroiliac joint synovitis at home and abroad, so far. Here we report a 34-year-old female patient who was diagnosed with PNVS by studying the clinical manifestations, imaging, histopathology, and pathology of the disease. The synovial membrane of the lesion was excised as thoroughly as possible, without radiotherapy or molecular targeted therapy before and after surgery. The patient was followed up for 2 years after surgery and has recovered well, with no related complications and no obvious tumor recurrence. The objectives of this passage are to discuss the diagnosis, differential diagnosis, treatment plan, invasive analysis, and prognosis of PNVS of the sacroiliac joint by reporting this case and reviewing relevant literature.

## Case Report

**Figure 1:**
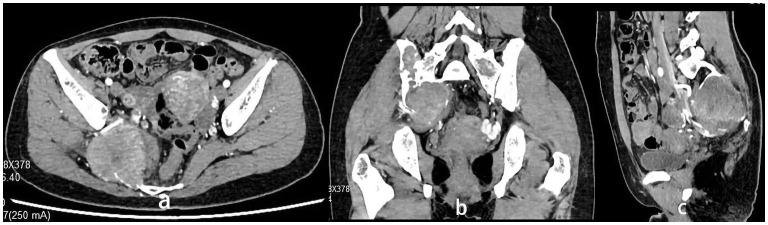
**(a–c)** Computer-enhanced tomography showing local bony expansion and destruction of the right sacrum and iliac crest, with areas of low-density liquefied necrosis within, and disappearance of the right sacroiliac joint space. CT showed an abundant blood supply of the tumor.

**Figure 2:**
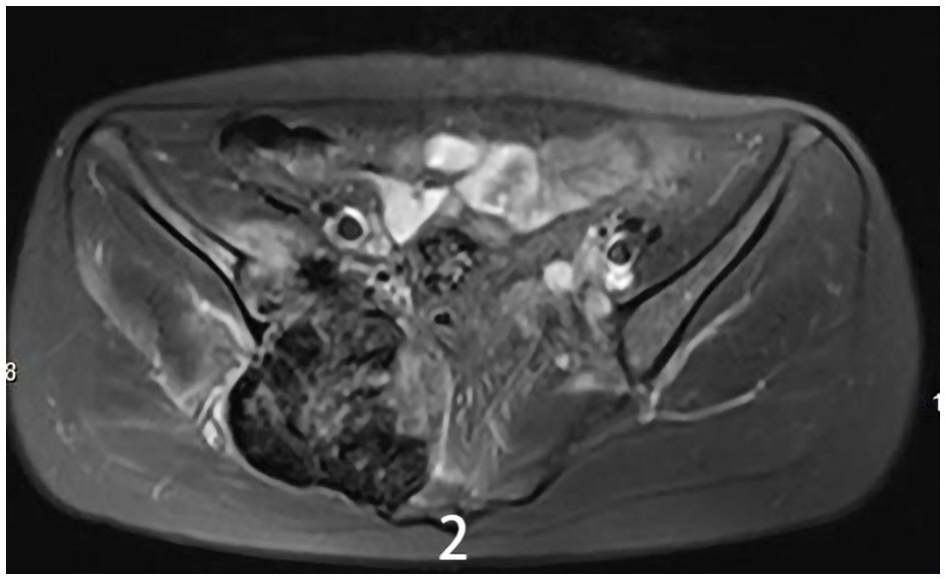
MRI showed a tumor of the right sacrum and the ilium boundary was still clear, the size of which was about 63 ^*^ 91 ^*^ 107 mm.

**Figure 3:**
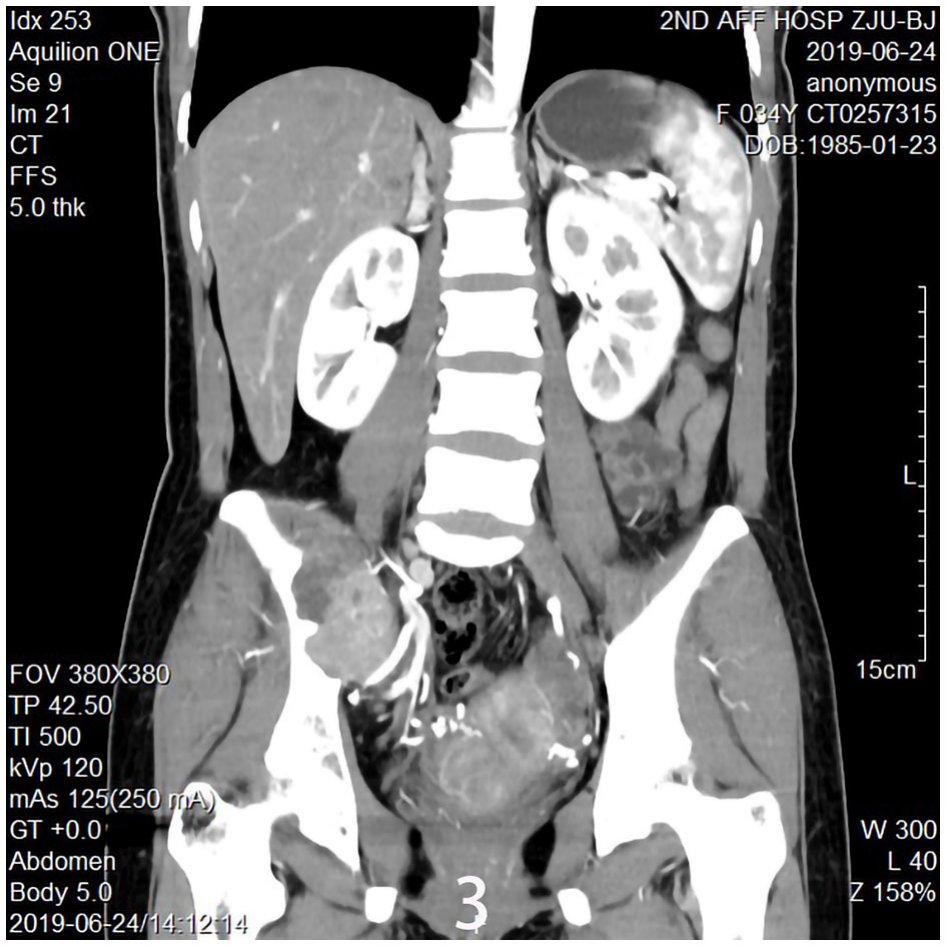
Imaging of the abdominal aorta shows the blood supply of the lesion came from the internal iliac artery.

The 34-year-old female patient of Han nationality, from Lanxi city, Zhejiang province, had no history of trauma or other diseases, and no family history of similar diseases. She went to the Second Affiliated Hospital of Zhejiang University for treatment. The chief complaint was sacroiliac joint pain with mild swelling for 10 months. The patient only took analgesic medicines orally and did not take other ways of treatment because the pain was not strong enough and only got stronger when doing strenuous activities. Computer-enhanced tomography showed local bony expansion and destruction of the right sacrum and iliac crest, with areas of low-density liquefied necrosis within, and disappearance of the right sacroiliac joint space (Figures 1a–c). MRI revealed a tumor in the right sacrum and ilium, the size of which was about 63 ^*^ 91 ^*^ 107 mm. The tumor boundary was still clear, and the texture inside the lesion was uneven, showing multiple cystic nodules. The lesion broke through the bone cortex and infiltrated into the adjacent tissue space, with a mild irregular response zone around the lesion (Figure 2). Imaging of the abdominal aorta showed that the blood supply of the lesion came from the internal iliac artery (Figure 3). Pathological results of preoperative puncture suggested pigments villonodular synovitis. Histological image showed diffuse + CD68, diffuse + CD163, Ki-67 10–15% +, partial + CD31, and other immune molecules were negative. Bone imaging of the whole body showed no obvious abnormality.

## Results

**Figure 4:**
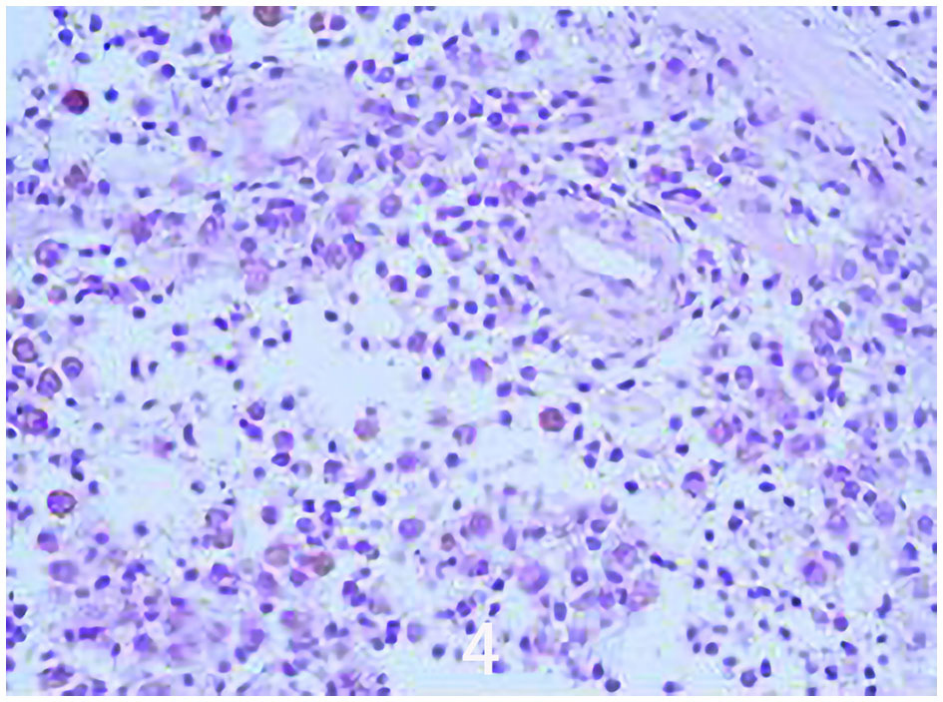
Histological image showing a tumor characterized by a cleft-like space growth lined by synovial-like cells and a mixed cellular component made-up of mononuclear round small and large discohesive cells, rare osteoclast-like giant cells, and inflammatory cells (Hematoxylin and Heosinstained (H & H) section; original magnification × 100).

**Figure 5:**
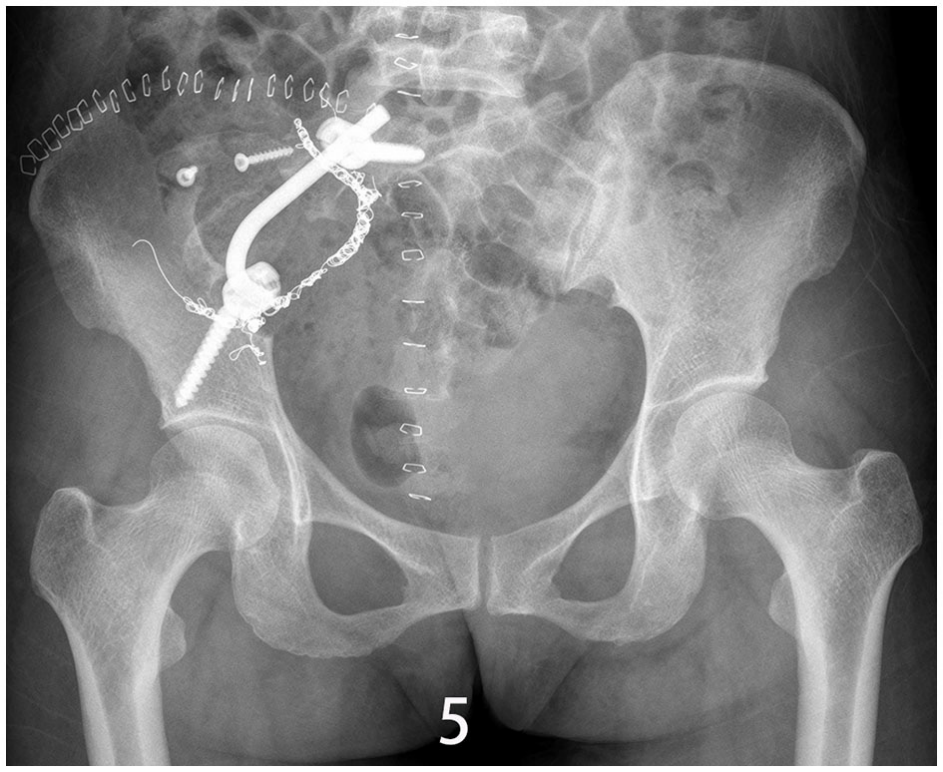
X-ray/CT image of the sacroiliac joint showing synovectomy and arthrodesis followed up 1.5 years after surgery.

**Figure 6:**
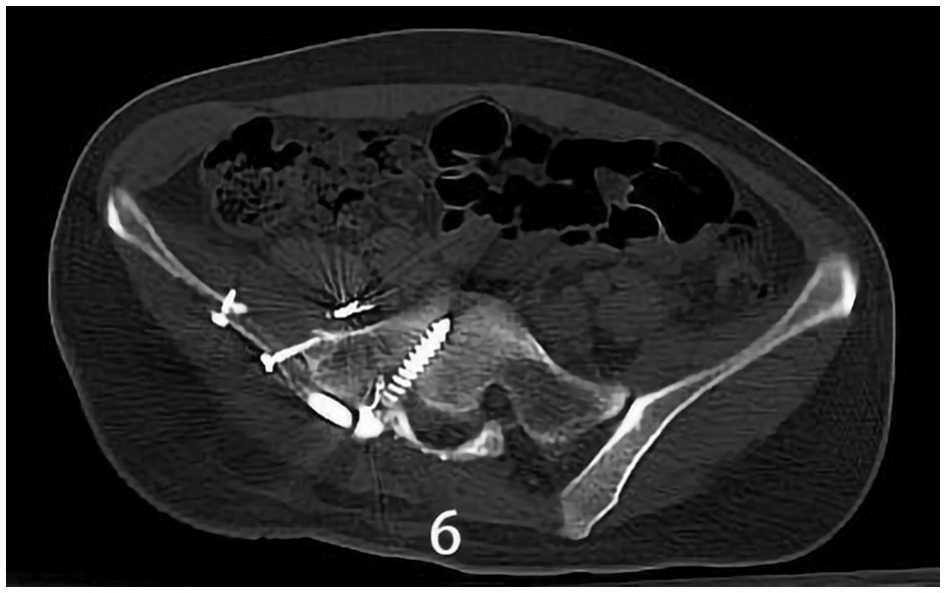
X-ray/CT image of the sacroiliac joint showing synovectomy and arthrodesis followed up 1.5 years after surgery.

The specific surgical treatment was as follows. To reduce intraoperative excessive blood loss and maintain a clear surgical field, preoperative abdominal aorta imaging was used to evaluate the tumor blood supply, and the main blood supply artery of the tumor was determined to come from the internal iliac artery. The tumor supplying artery embolization with bilateral iliac arteriography has been done 1 day before surgery with the help of the interventional department. After the successful operation of general anesthesia, the patient was placed in the prone position. After routine disinfection and towel laying, we used the posterior incision of the pelvic tumor: an incision of about 20 cm in length was made centering on the sacrum, and an incision of about 15 cm was made from the level of the iliac crest to the right. The skin, subcutaneous tissue, and fascia were cut sequentially, and the muscle and soft tissue were stripped from the spinous process to the right. Intraoperatively, a large tumor of the sacrum was observed, which destroyed the sacroiliac joint and enveloped the right S2 nerve root. The soft tissue mass in front and behind the sacroiliac joint was prominent. The space between the gluteus maximus and the tumor capsule was separated to the greater sciatic notch to expose and protect the S1 nerve root and the sciatic nerve, and the lesion was gradually separated to the tumor boundary and the integrity of the tumor capsule was maintained. Since the S2 nerve root was surrounded by the tumor, the tumor was segmented to separate and protect the S2 nerve root along the S2 nerve root. After the incision was rinsed with a large amount of normal saline, a pedicle screw was placed in S1, and an iliac screw was placed in the right iliac bone. Intraoperative X-ray fluoroscopy indicated that the internal fixation position was good, the prebent fixation rod was installed, and the nut was tightened. The iliac bone grafts were taken from the posterior iliac bone between the sacrum and the iliac bone, and internal fixation was performed with screws. The ideal placement of the internal fixation was confirmed by X-ray enhanced fluoroscopy again. A large amount of normal saline was rinsed again, and the bleeding was stopped completely. After the instruments and gauze were checked without any problems, a drainage tube was placed, and the incision was closed by a layer-by-layer suture. The patient lost 1,200 ml of blood and was given the infusion of 1,100 ml suspended red blood cells and 1,000 ml of plasma. The pathological diagnosis after the operation was PNVS (Figure 4). After the operation, the patient lay in bed for 1 month, followed by low-molecular heparin anticoagulation for 1 month, and gradually recovered. After a 2-year follow-up, the patient did not receive radiotherapy or molecular therapy, and no tumor recurrence was confirmed by Computer-enhanced tomography/MRI (Figures 5, 6), and the joints of the patient function well.

## Discussion

The etiology and pathogenesis of PVNS remain unclear, and the relationship between traumatic history and PVNS remains controversial. Some scholars have proposed that most PVNS have CSF1 gene translocation on chromosome 1p13, which will lead to overexpression of CSF1 and, thus, promotes tumor progression (4). The current consensus is that it is due to a combination of inflammation and tumor. At present, PVNs are mainly characterized by extensive synovial hyperplasia and erosion of inflammatory factors, and the long-term existence of these inflammatory cells will lead to repeated joint cavity bleeding, subchondral cystic degeneration, and bone erosion (5).

Patients with pigmented villonodular synovitis with sacroiliac joints initially presented only mild pain and swelling on the side of the buttocks. The tumor did not directly involve the sacral nerve, so the patients had no lower limb motor and sensory dysfunction. These early clinical symptoms are not obvious signs of the disease, which may lead to missed diagnosis and misdiagnosis. The proportion of patients with PVNS with normal or possible non-specific soft tissue swelling on X-ray examination was 54% (6).CT can clearly show bone destruction area and low-density liquefaction necrotic area. Meanwhile, a CT-guided synovial biopsy can be further performed to confirm the diagnosis. MRI examination is the best non-invasive method for the diagnosis of PNVS (7). On MRI, villous nodules show marked hypo-intensity changes at T 2 W I, which are characteristic of pigmented villous nodular synovitis. This is due to the high content of hemosiderin in the villous nodules, whose paramagnetism significantly reduces the T 2 relaxation time. MRI examination of PVNs of the sacroiliac joint showed mixed low signals in the T1-weighted images, while mixed high and low signals in the T2-weighted images, which exactly indicated that the occurrence of high signals was related to inflammation, edema, necrotic components, and lipid components in the nodules (8).

According to the six MRI parameters defined by Mastboom et al. (9) and the classification of the severity of pigmentation villonodular sacroiliac synovitis through the MRI scan results of 118 patients treated for giant cell tumor of the tendon sheath by Leiden University Medical Center (LUMC), the MRI findings, in this case, were more invasive. At the same time, MRI is also an important auxiliary examination for detecting surgical effects, tracking tumor recurrence, and determining the presence or absence of osteonecrosis. Full abdominal aortic imaging is a necessary and complete examination before surgery for pigmented villonodular synovitis of the sacroiliac joint, which is determined by the particularity of the anatomical structure of the sacroiliac joint and aims to determine the blood supply source of the tumor. A synovial biopsy is a gold standard for the diagnosis of PNVS. Alexiev et al. classified pigmentation villonodular synovitis into benign and malignant, and the classification basis mainly depended on the histological characteristics and immunohistochemistry of the tumor (10). Bertoni et al. (11) reported eight cases of malignant PVNS early in 1997 (three cases were secondary and five cases were primary) and described the histological characteristics of malignant PVNS. In this case of PNVS of the sacroiliac joint, monocytes or histiocytoid cells with pigmentation were found histologically, but none of the above malignant histological features were observed. Immunohistochemistry showed diffuse + CD68, diffuse + CD163, and partial + CD31, suggesting that the diseased cells were of tissue origin and Ki-67 was 10–15% +. Combined with the imaging and histological and immunological examinations of the patient, the PVNS of the sacroiliac joint in this case was a benign tumor with strong aggressiveness and easy recurrence. So far, six cases of benign PVNS with metastasis have been described in the foreign literature (see the chart below), thus, it can be seen that patients with benign PVNS need to undergo whole-body bone imaging or whole-body glucose metabolism imaging to detect metastases, and no abnormalities were found in this patient.

The PVNS of the sacroiliac joint mainly needs to be differentiated from synovial sarcoma, synovial osteochondromatosis, joint tuberculosis, hemophiliac osteoarthritis, osteoarthritis, joint amyloidosis, joint infectious inflammation, and rheumatoid arthritis. Among them, the imaging examination of sacroiliac joint pigmentation villonodular synovitis is likely to be malignant, so it is difficult to distinguish it from synovial sarcoma. There is calcification in synovial sarcoma accompanied by periosteum reaction, and the identification mainly depends on pathological examination.

Pigmented villonodular synovitis is a benign tumor with local invasive characteristics (12), with surgery as the preferred treatment method, and intraoperative adequate resection of tumor tissue and pathological synovium is the key (13). Since the boundary of the sacroiliac joint pigmented villonodular synovitis is still clear in this case, we treated the patient with surgery to completely remove the disordered synovitis. For the knee and hip joint, prosthesis replacement technology is becoming more and more mature, and arthroplasty can be performed for severe joint damage or evidence of recurrent malignancy. However, the activity of the sacroiliac joint is very small, about 3–5°, surrounded by many strong ligaments, which are conducive to supporting weight and transmitting gravity. As a special joint with almost no joint activity, pigmented villonodular synovitis is often accompanied by severe bone destruction. Therefore, open surgical treatment of disaffected tissue resection and internal fixation with bone graft seems to be the only treatment to achieve a good prognosis. Preoperative embolization of tumor blood supply is beneficial to maintaining the integrity of the tumor envelope. Local masses can usually be completely removed, while for diffuse masses, complete removal of the tumor is often more difficult. It has been reported that the postoperative local control rate of PVNS alone is 44~92% (14). Blanco et al. suggested that adjuvant therapy for diffuse PVNS (15) can remove the remaining villous nodular cells and reduce the recurrence rate. Among the current treatments, most scholars recommend radiotherapy as an adjunct to total synovectomy to prevent recurrence in residual tumors or as a second-stage treatment for recurrence. As a surgeon, we should consider the side effects brought by radiotherapy: 1. Combined with a large amount of bone destruction, or incomplete healing of bone graft, radiotherapy can aggravate bone destruction or lead to delayed healing and nonunion of bone graft, or even radionecrosis. 2. Fatigue, nausea, loss of appetite, leukopenia, and other symptoms. 3. Acute radiation damage leads to local skin ulcers, bleeding, and necrosis. 4. A large amount of joint effusion, swelling, stiffness, pain aggravation, radioactive synovitis. 5. In the reports of malignant PVNS, there is usually a history of radiotherapy (16), which can lead to sarcomatosis, which may occur simultaneously within 7 years after treatment. In terms of molecular therapy, Dewar et al. (17) published studies on the inhibition of macrophage (M) -CSF receptor (CS F-1R) and c-FMS with imatinib. Imatinib, as a tyrosine kinase inhibitor, has been shown to block the BCR-ABL complex, PD GFR, c-kit proto-oncogene, ABL, and ABL-related genes (17, 18). Early data showed that CSF1R inhibitors did not cure most patients, and some patients showed significant responses (fatigue, nausea, dermatitis, edema, and myelosuppression) in the first few months of treatment, but eventually symptoms were alleviated (19). For patients with multiple metastases and metastatic disease, systemic treatment with CSF1 inhibitors may improve the incidence (5). Therefore, the therapeutic role of molecular therapy in PVNS still requires more research to understand the true efficacy, long-term side effects, and optimal treatment time of targeted drugs.

In the current WHO classification, PVNS is considered to be locally invasive and non-metastatic tumors with uncertain behavior (20). Malignant PNVS is very rare, and pathologists indicate that it may exist alone as a primary malignancy, coexist with benign neoplasms, or develop from benign malignancies. Data from a study showed that distant metastasis of malignant PNVS mainly occurred in lung and lymph nodes (11)^.^Although PVNS are benign tumors, they are invasive. To date, 6 cases of metastatic benign PVNS have been described in the literature (Table 1) (1, 21–25). This table demonstrates that metastatic aggressive PVNS can occur and that metastases usually occur after several local recurrences, with a duration of 5 to 49 years after the initial surgery. For benign PVNS, there may be metastasis, which is often a problem that we tend to ignore.

**Table 1:** The reported case of histologically benign diffuse tenosynovial giant cell tumor (TS-GCT) developing metastases.

					**Metastases**			
**Authors**	**Sex/age**	**Tumor location**	**Subtype**	**Site**	**Previous local recurrence (times)**	**Duration from primary surgery (years)**	**Treatment**	**Clinical Site outcome**
Choong et al. (21)	F/35	Popliteal fossa	Intra-articular	Soft tissue	5	49	NA	NA
Somerhausen and letche.(1)	M/50	Popliteal fossa	Extra-articula	Lymph nodes	2	5	NA	NA
Sikaria et al.(22)	M/49	Wrist and forearm	Extra-articular	Lung, lymph nodes	4	9	CT	DOD
Asano et al.(23)	M/45	Proximal tibiofibular joint	Intra-articular	Lung, soft tissue, lymph nodes	1	8	None	AWD
Righi et al.(24)	M/38	Knee joint	Intra-articular	Lymph nodes	3	25	CT	AWD
Osanai et al.(25) (present case)	F/41	Buttock	Extra-articular	Lung, soft tissue, subcutis	0	0	RT	DOD

*TS-GCT, tenosynovial giant cell tumor; CT, chemotherapy; RT, radiotherapy; NA, not available; DOD, died of disease; AWD, alive with disease*.

## Conclusion

Pigmented villonodular synovitis occurs not only in the knee joint, hip, ankle, wrist, shoulder joint, small joint of the hand and foot but also in the sacroiliac joint. Because the early clinical symptoms and signs are not obvious, it is easy to miss diagnosis and misdiagnosis. Full abdominal aorta imaging is the necessary routine examination for the sacroiliac joint PVNS preoperative. MRI is the best non-invasive method to diagnose PVNS, but the diagnosis depends on pathological biopsy. Pigmented villonodular sacroiliac joint synovitis is more invasive, and may be more prone to recurrence and metastasis. In terms of treatment, surgery is the first choice to completely remove the synovitis as much as possible, and bone grafting and fusion internal fixation are the first choices to stabilize the sacroiliac joint when bone damage is serious. Because the side effects and therapeutic effects of radiotherapy and molecular targeted therapy are uncertain, separate, and postoperative radiotherapy and molecular targeted therapy are not recommended. Since surgery has a high recurrence rate, we will re-evaluate the possibility of surgical resection in cases with recurrence; given the possible occurrence of PVNS metastasis or malignant transformation, we will continue to follow up on the case. In the face of the ever-changing and booming medical technology, there will certainly be better ways to help us solve the current medical problems in the future.

## Data Availability Statement

The original contributions presented in the study are included in the article/supplementary material, further inquiries can be directed to the corresponding author.

## Ethics Statement

The studies involving human participants were reviewed and approved by Human Research Ethics Committee of the Second Affiliated Hospital of Zhejiang University Medical College. The patients/participants provided their written informed consent to participate in this study. Written informed consent was obtained from the relevant individual for the publication of any potentially identifiable images or data included in this article.

## Author Contributions

BL: operation and manuscript. JS: manuscript and literature. KJ and ZL: follow-up. All authors contributed to the article and approved the submitted version.

## Funding

This research was supported by Grants from the National Natural Science Foundation of China (NO. 81872181) and Natural Science Foundation of Zhejiang Province (NO. LY21H160034).

## Conflict of Interest

The authors declare that the research was conducted in the absence of any commercial or financial relationships that could be construed as a potential conflict of interest.

## Publisher's Note

All claims expressed in this article are solely those of the authors and do not necessarily represent those of their affiliated organizations, or those of the publisher, the editors and the reviewers. Any product that may be evaluated in this article, or claim that may be made by its manufacturer, is not guaranteed or endorsed by the publisher.
